# CRISPR/Cas9-mediated genome editing in wild-derived mice: generation of tamed wild-derived strains by mutation of the *a (nonagouti*) gene

**DOI:** 10.1038/srep42476

**Published:** 2017-02-14

**Authors:** Michiko Hirose, Ayumi Hasegawa, Keiji Mochida, Shogo Matoba, Yuki Hatanaka, Kimiko Inoue, Tatsuhiko Goto, Hideki Kaneda, Ikuko Yamada, Tamio Furuse, Kuniya Abe, Yoshihisa Uenoyama, Hiroko Tsukamura, Shigeharu Wakana, Arata Honda, Atsuo Ogura

**Affiliations:** 1 RIKEN BioResource Center, Tsukuba, Ibaraki 305-0074, Japan; 2 Graduate School of Life and Environmental Science, University of Tsukuba, Ibaraki 305-8572, Japan; 3 Department of Biological Production Science, College of Agriculture, Ibaraki University, Ami, Ibaraki 300-0393, Japan; 4 Graduate School of Bioagricultural Sciences, Nagoya University, Nagoya, Aichi 464-8601, Japan; 5 Organization for Promotion of Tenure Track, University of Miyazaki, Miyazaki 889-1692, Japan; 6 The Center for Disease Biology and Integrative Medicine, Faculty of Medicine, University of Tokyo, Tokyo 113-0033, Japan

## Abstract

Wild-derived mice have contributed to experimental mouse genetics by virtue of their genetic diversity, which may help increase the chance of identifying novel modifier genes responsible for specific phenotypes and diseases. However, gene targeting using wild-derived mice has been unsuccessful because of the unavailability of stable embryonic stem cells. Here, we report that CRISPR/Cas9-mediated gene targeting can be applied to the Japanese wild-derived MSM/Ms strain (*Mus musculus molossinus*). We targeted the *nonagouti (a*) gene encoding the agouti protein that is localized in hair and the brain. We obtained three homozygous knockout mice as founders, all showing black coat colour. While homozygous knockout offspring were physiologically indistinguishable from wild-type litter-mates, they showed specific domesticated behaviours: hypoactivity in the dark phase and a decline in the avoidance of a human hand. These phenotypes were consistent over subsequent generations. Our findings support the empirical hypothesis that *nonagouti* is a domestication-linked gene, the loss of which might repress aggressive behaviour.

The mouse (*Mus musculus*) is the most frequently used mammalian species for biomedical research because of its defined genetic background and the relative ease of carrying out genetic modification. Since the 1980s, gene-targeting strategies using embryonic stem cells (ESCs) have made this species the standard experimental model for understanding gene functions and associated disease mechanisms^1^. However, the genetic diversity of classical laboratory mouse strains is thought to be limited because of closed breeding using genetically related ancestral strains^2^. Furthermore, such domesticated mice are more prone to many types of tumours and metabolic diseases, such as diabetes, when compared with other species^1^. Therefore, it is desirable to use genetically diverse strains of mice including wild-derived mice for pursuing further genetic and physiological studies. Wild-derived strains are relatively introduced genetic resources and are expected to increase the chance of finding novel modifier genes that are responsible for some disease-resistance or disease-prone phenotypes. Thus, we expect that they might provide invaluable information on corresponding diseases in humans^3^,^4^,^5^. Furthermore, a large pool of polymorphisms that discriminate conventional laboratory mice from wild-derived mice might enable more accurate and more efficient quantitative trait locus (QTL) analyses^6^,^7^. However, targeted genetic modifications in wild-derived strains have been more difficult to achieve in comparison to laboratory strains because they are generally resistant to assisted reproductive technologies (ARTs). This is also compounded by the unavailability of stable ESC lines, which have been the main tools for generating targeted genetic modifications. We have recently devised basic ART methods including superovulation, *in vitr*o fertilization (IVF), embryo transfer, and sperm/embryo cryopreservation for wild-derived strains in different subspecies of *Mus musculus*^8^. Many wild-derived strains can now be cryopreserved and bred safely using these designated ARTs at our centre and other mouse repository facilities^9^.

Recently developed genome-editing technologies are contributing significantly to biomedical research by generating animals or cell lines carrying expected mutations. Zinc finger nucleases (ZFNs), transcription activator-like effector nucleases (TALENs), and clustered regularly interspaced short palindromic repeats (CRISPR)/Cas9 have already been applied successfully to gene targeting in laboratory mice^10^,^11^. One of their most important advantages is that gene-targeted mice can be generated without the intervening steps of generating ESCs and germ line chimaeras. However, there have been no reports on genome-editing approaches in wild-derived mice to date. Here, we employed the CRISPR/Cas9 system to target the *nonagouti (a*) gene that encodes the agouti protein using a Japanese wild-derived mouse strain, MSM/Ms. This strain belongs to a subspecies (*Mus musculus molossinus*) of the laboratory mouse (*M. m. domesticus*) and has been established by Dr Kazuo Moriwaki as a strain with a defined genetic background produced by inbreeding^12^. We found that the generation of gene knockout (KO) MSM/Ms mice by the CRISPR/Cas9 system was highly practical. Furthermore, phenotypic analysis of the mutant mice provided the first experimental evidence of the *nonagouti* gene as a domestication-linked gene.

## Results

### Generation of *nonagouti* KO lines

**Figure 1:**
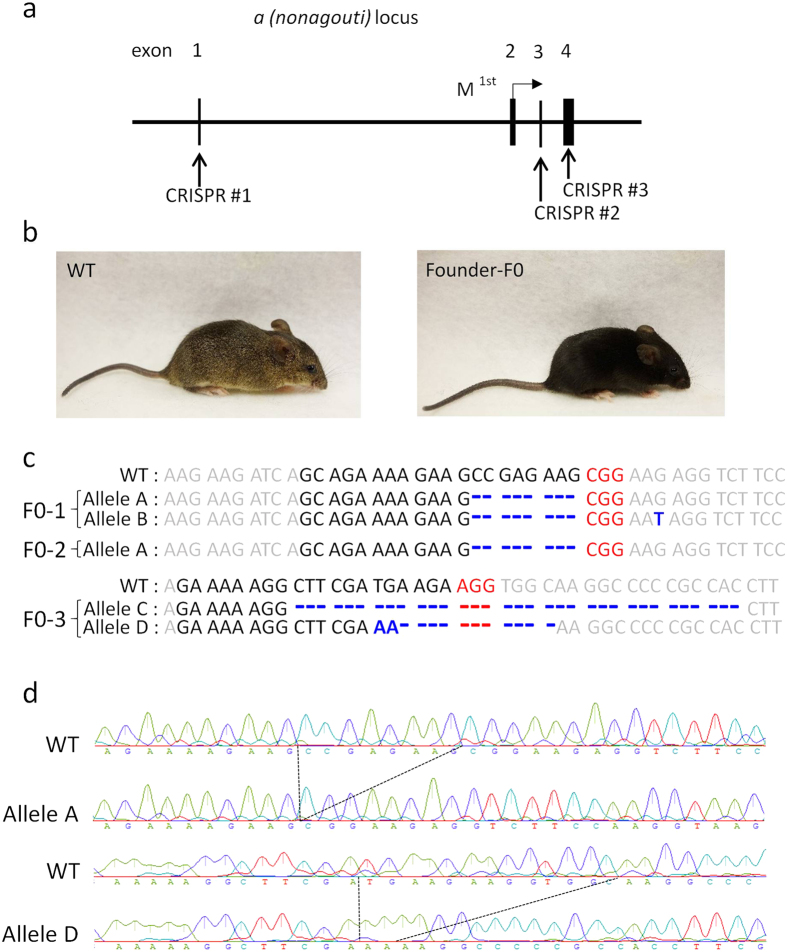
The *nonagouti (a*) mutation in MSM/Ms mice. (**a**) Schematic representation of sgRNA targeting *nonagouti (a*). (**b**) Founder (F0) knockout (KO) and wild-type (WT) mice. The F0 mice had black coats, indicating loss of the agouti protein. (**c**) DNA sequencing analysis of the CRISPR target site in the MSM/MS WT and founder mice. Indels (insertions, blue bold characters; deletions, dashed lines) were identified at the sgRNA targeted loci. Black and red bold characters represent sgRNA recognition sites and PAM sequences, respectively. Four mutated alleles (A to D) were observed in the founder KO mice. (**d**) DNA sequencing analysis of homozygous KO mice (F2) from lines A (upper) and D (lower).

*In vitro*-transfection experiments using HEK 293 cells showed that each sgRNA sequence had high efficacies of double-strand break (DSB)-mediated homology-dependent repair (Supplementary Fig. S1). The targeting constructs were injected into a total of 127 zygotes and 92 (72%) of these developed into 2-cells during 24 h culture (Table 1). After being transferred into recipient pseudopregnant female mice, three male pups were born alive at term: none, two, and one from three different target sites (CRISPR #1, #2, and #3), respectively (Fig. 1a and Table 1). All three offspring had black coat colour, which indicated that they carried biallelic conversions (Fig. 1b). Genomic sequencing analysis around the target sites confirmed biallelic conversions in these mice and identified four types of mutant alleles, which we tentatively named alleles A, B, C, and D (Fig. 1c). Alleles A, B, C, and D had 8, 8, 33, and 11 (−13 + 2) nucleotide deletions, respectively. Therefore, in alleles A, B, and D, the coding regions of the gene may create frame shift mutations that result in loss-of-function. After mating these founder (F0) mice with wild-type (WT) MSM/Ms mice and intercrossing of the resultant heterozygous F1 mice, we generated two KO lines, each carrying alleles A or D. The sequences of the homozygous mutations in the two KO lines of the F2 generation are shown in Fig. 1d. The knockout alleles could also be identified by genomic PCR (Supplementary Fig. S2). We confirmed that there was no integration of the vector sequences in the genome of the KO lines (Supplementary Fig. S3). The mice showed no fertility problems and the mutant alleles were segregated at a normal Mendelian ratio. Then, lines A, D, or both were subjected to a series of phenotypic analyses at defined ages, as described below.

### Physiological parameters

A series of physiological parameters were analysed at defined ages according to the pipeline for the fundamental screening at the Japan Mouse Clinic, RIKEN (http://ja.brc.riken.jp/lab/jmc/mouse_clinic/en/) using lines A and D. At 8 weeks of age, mice were analysed by modified SHIRPA, a protocol for comprehensive phenotype screening^13^. There were no genotype-related differences except for a few minor differences including the body mass index and the tail/body length ratio (line A) and body length (line D) (*P* < 0.05) (see Supplementary Fig. S4). At 19 weeks, mice were examined for serum biochemical parameters and serum adipocytokines. There were no differences between WT mice and homozygous KO mice in the clinical parameters analysed in serum except for high-density lipoprotein (HDL) cholesterol in female mice of line A (*P* < 0.05) (see Supplementary Fig. S5). At 26 weeks of age, mice were euthanized by cervical dislocation and were examined for the gross morphology of organs. No genotype-related differences were observed in all parameters examined including the adrenal gland weight (*P* > 0.05), which is increased in rats with aggressive behaviour^14^ (see Supplementary Fig. S6).

### Behavioural analysis

**Figure 2:**
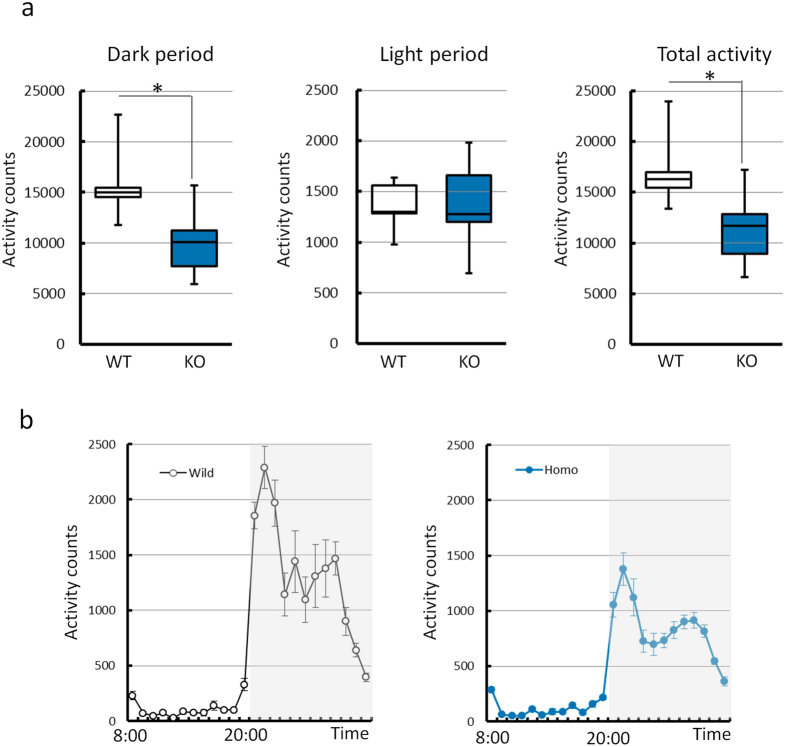
Comparison of the amount of activity in the home cage activity test. (**a**) The amount of activity observed with wild-type (WT) and knockout (KO) mice in the dark period (left), light period (middle), and in the total period (right). The activity counts in the dark and total periods were lower in the KO mice than WT mice. *P < 0.05 (WT vs. KO mice, *t* test) (**b**) Mean amount of activity per hour over 5 days. White and grey boxes show the light and dark periods, respectively. The data were obtained from mice from line D (5 WT mice and 13 KO mice) and are presented as the mean ± S.E.M.

Animals were analysed for behavioural tests at defined ages according to the pipeline for fundamental screening at the Japan Mouse Clinic, RIKEN. There were no genotype-related differences in the results of the light/dark transition test (8 weeks), first open-field test (9 weeks), and second open-field test (10 weeks) in either sex. In the home cage activity test, the KO mice displayed relative hypoactivity in the dark phase and over a 24 h period compared with WT litter-mates (*t* test; *P* < 0.05). The activity counts per hour indicated that the reduction in activity in the dark phase (20:00–08:00) was caused by decreased activity in the whole of this phase, and was not limited to specific periods (Fig. 2).

### Tameness test

**Figure 3:**
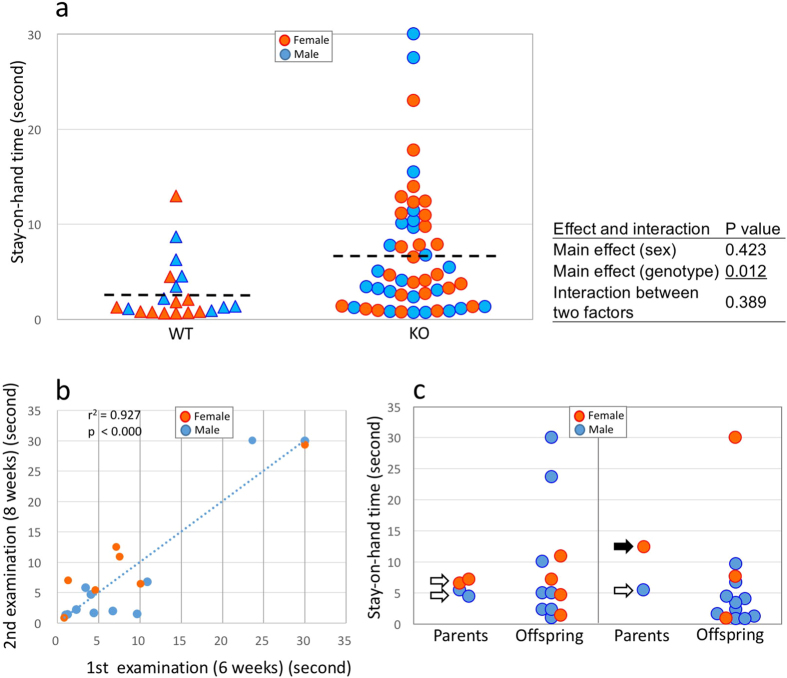
Results of the stay-on-hand test. (**a**) Comparison of the stay-on-hand test in wild-type (WT) mice and *nonagouti (a*) knockout (KO) mice. Genotype, but not sex, had a significant effect on the duration of time on the hand and there was no interaction between these two factors (two-way ANOVA). The dashed lines represent the average values. (**b**) Correlation of stay-on-hand duration traits between the first (6 weeks) and second (8 weeks) examinations. (**c**) Correlation of stay-on-hand duration traits between parents and their offspring. The left panel shows the offspring of moderate duration × moderate duration parents; the right panel shows the offspring of moderate duration × long duration parents. The open and black arrows indicate moderate and long durations, respectively. The data were obtained from mice of line A.

At 6 weeks of age, animals were examined for tameness by the stay-on-hand test. Genotype, but not sex, had a significant effect on the duration that the mice remained on the hand (two-way ANOVA, *P* < 0.05). There was no significant interaction between these two factors (two-way ANOVA, *P* > 0.05). This indicated that the homozygous KO mice stayed on a human hand for longer than WT mice (Fig. 3a and Supplementary Movies S1 and S2). Then, the animals were examined again at 8 weeks to determine whether these tendencies were specific for each individual or appeared at random on the day of the experiment. As shown in Fig. 3b, a high correlation was observed between the results of the first and second examinations, indicating that the quantitative trait of the stay-on-hand test was stable in individual animals. We next examined whether the quantitative trait of the parents would be carried over to their offspring. We tested two parental combinations according to their phenotypes in terms of the duration of stay in this test: moderate × moderate, and moderate × long. We found that the duration of staying on a human hand in their offspring showed variations that were irrespective of the combination of parents (Fig. 3c). The mean and variance of the time of staying on a hand were not significantly different between the two parental combinations (*t* test and F test, respectively). This finding indicated that although this quantitative trait might be variable, this tameness tendency was genetically fixed in this strain.

### Gene expression and biochemical analysis of the midbrain

**Figure 4:**
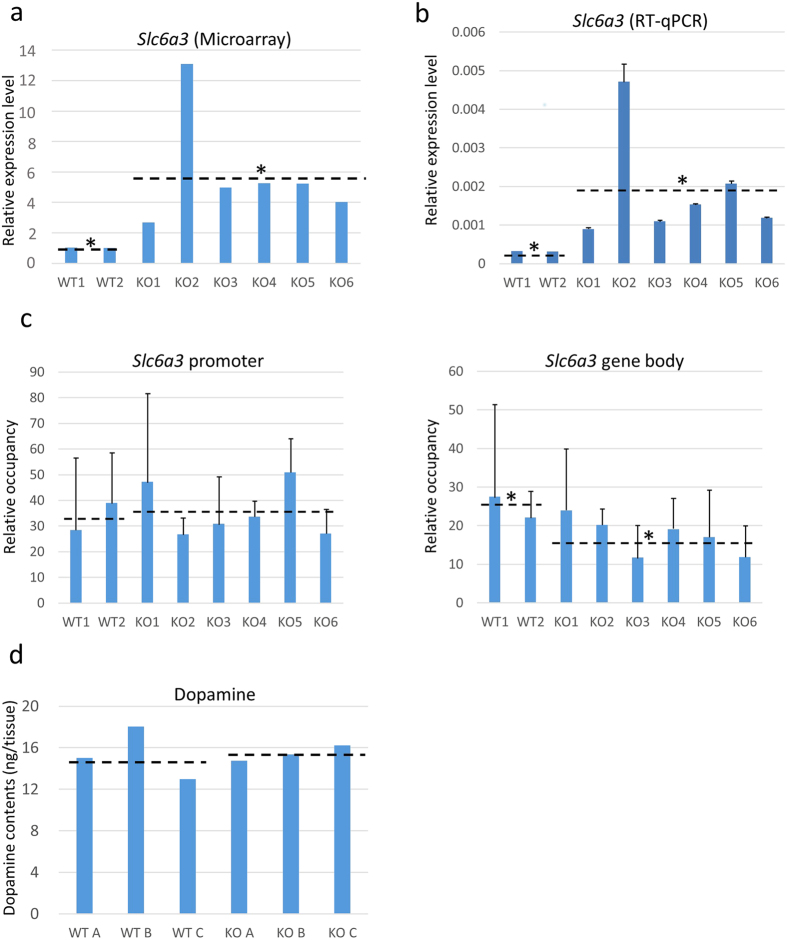
Levels of gene expression and H3K27me3 enrichment in the *Slc6a3* gene, and dopamine concentrations in the midbrain. (**a**) The expression levels of *Slc6a3* measured by microarray. The average of wild-type (WT) samples was set as 1. *P < 0.05 (WT vs. knockout (KO) mice, *t* test). (**b**) The expression levels of *Slc6a3* measured by RT-qPCR analysis. The mean level of WT was set as 1. *P < 0.05 (WT vs. KO mice, *t* test). Data are presented as the mean ± S.D. (**c**) ChIP analysis of enrichment of H3K27me3. There was a significant difference in the body of *Slc6a3*, but not in the promoter region. *P < 0.05 (WT vs. KO mice, *t* test). See Materials and Methods for the calculation method of the relative occupancy. Data are presented as the mean ± S.D. (**d**) Dopamine concentrations in the midbrain. There were no genotype-related differences. All data were obtained from mice of line A. The dashed lines represent the average values of each group.

To gain clues to understanding the physiological or biochemical basis underlying the domesticated behaviour of *nonagouti*-deficient MSM/Ms mice, we undertook gene expression analysis on midbrain samples using a microarray. Out of 34,469 probes detected with reliable levels of signal intensity, 379 and 200 showed more than twofold up- and down-regulation, respectively, in homozygous KO mice compared with WT mice (Supplementary Table S1). We searched for genes related to neurotransmitters and found that the mRNA level of *Slc6a3*, a dopamine transporter gene, was significantly higher in homozygous KO mice than WT mice (*t* test; *P* < 0.05) (Fig. 4a). Our quantitative reverse transcription PCR (RT-qPCR) analysis further confirmed this genotype-dependent difference in the *Slc6a3* level (*t* test; *P* < 0.05) (Fig. 4b). Other neurotransmitter-related genes did not show differential expressions between KO mice and WT mice by microarray analysis (Supplementary Fig. S7). Kyoto Encyclopedia of Genes and Genomes (KEGG; http://www.genome.jp/kegg/) pathway analysis showed that 379 up-regulated genes included eight pathways that correlated with dopamine transmission or dopaminergic synapse (shown with red characters in Supplementary Table S2). Of these pathways, four included *slc6a3* in their components (red characters with asterisks in Supplementary Table S2). GO analysis also showed that up-regulated genes are significantly involved in neurotransmitter biosynthetic or transport processes (e.g. GO:0042416 dopamine biosynthetic process, GO:0042165 neurotransmitter binding, red characters in Supplementary Table S3). Taken together, up-regulated genes in KO mice may have a strong effect on their behaviour by the alteration of neurotransmitter transport or syntheses.

We then undertook chromatin immunoprecipitation analysis of any histone modifications on the *Slc6a3* gene because social isolation stress in mice was reported to change the histone modification pattern at the *Htr5b (5-HT receptor 5B*) gene^15^. We found that the gene body of *Slc6a3* contained a significantly lower density of histone H3-Lys27 trimethylation (H3K27me3) in homozygous KO mice compared with WT mice (*t* test; *P* < 0.05) (Fig. 4c). The patterns of H3K4me3 and H3K9me3 enrichment were highly variable. Finally, we compared the dopamine content in the midbrain tissues from homozygous KO and WT mice, but there was no genotype-dependent difference (Fig. 4d).

## Discussion

The CRISPR/Cas9-mediated gene-editing system is a robust method for loss-of-function studies in mammals including mice^16^. Here, we first attempted to apply this system to gene KO of wild-derived strains of mice. We chose the *nonagouti* gene as the target gene because homozygous mice can be easily identified by their coat colour. The *nonagouti* gene encodes the agouti protein, which alternates the production of light and dark pigments on a single hair as it grows, creating different colour bands: the “agouti” coat colour^17^. The most typical phenotype of a mutation of the *nonagouti* gene is the black coat colour, as observed in the C57BL/6 mouse strain. The efficiency of KO by CRISPR/Cas9 was high in wild-derived mice; all three offspring carried homozygous KO alleles and, as expected, they showed a black coat colour. Thus, our study indicated that the CRISPR/Cas9 system combined with a series of ARTs specialized for wild-derived mouse strains^8^,^9^ could promote the generation of new animal models for studies on gene functions and relevant human diseases, which have not been attainable using conventional laboratory mice.

Domestication of animals may occur as a result of genetic adaptation to captivity and many genes or genetic regions have been identified as domestication genes/regions by QTL analysis^18^. The genetic processes with the greatest potential impact on the domestication are inbreeding, genetic drift and selection. Whereas the changes caused by selection are directional, inbreeding and genetic drift, produce random changes in gene frequencies^19^. The MSM/Ms strain we used has a relatively short inbreeding history and thus retains many traits related to behavioural wildness, including high aggressiveness and avoidance of humans^12^. Interestingly, a significant proportion of our KO MSM/Ms mice exhibited domesticated behaviour, as shown by the home cage activity test and the stay-on-hand test. Historically, the *nonagouti* gene has been thought to be one of the domestication genes because animals carrying a mutation of this gene—such as black foxes, mink, and deer mice—tend to exhibit tame behavioural patterns when exposed to humans^20^,^21^,^22^,^23^. However, it has been difficult to obtain compelling experimental evidence for this empirical hypothesis because of the lack of large cohorts of wild animals for analysis and their heterogeneous genetic backgrounds that could perturb the results. In this regard, the genetically defined MSM strain with aggressive behaviour could be an ideal experimental model for this purpose. In this study, we determined statistically that the deletion of the *nonagouti* gene could induce domesticated behavioural phenotypes in the MSM strain. In this study, although we used one of the two KO lines for each behavioural analysis or tameness test, we confirmed that both lines showed essentially similar tendencies at least for the behavioural analysis. As far as we know, this is the first experimental evidence for *nonagouti* to be a domestication-linked gene.

However, at present, we do not know exactly why the loss of the agouti protein induced tamed behaviour in MSM/Ms mice. The agouti protein is also found in the brain and is an antagonist of the melanocyte-stimulating hormone (MSH) at its receptor in neural tissues^24^,^25^,^26^. Gene expression analysis by microarray and RT-qPCR revealed that the *Slc6a3* gene, a dopamine transporter gene, was significantly more up-regulated in the midbrain of KO mice than in that of WT mice. Consistent with this, the gene body of the *Slc6a3* gene contained a low density of histone H3K27me3, a repressive epigenetic histone modification^27^. Meanwhile, we did not find any differences in the total dopamine levels in the midbrain between the KO and WT mice. It is possible that dopamine neurotransmission efficiency in the KO brains might have changed in response to increased local dopamine transporter activity, without changing the total amount of dopamine. Although this scenario needs further validation, there are reports on the close relationships between MSH and the dopamine system in the midbrain of several species^28^,^29^,^30^,^31^,^32^.

The *nonagouti* KO MSM/Ms strains we established will provide an invaluable model for the study of domestication and behaviour. At present, the degree of tameness in KO MSM mice cannot be controlled precisely, but the tamed behaviour is apparently a result of a genetic effect. Animal domestication has a long history and has attracted much research attention. The fact that only a single mutation was able to cause tamed behaviour should be advantageous for the identification of other related domestication factors. Furthermore, we emphasize that the mutated allele did not affect any systemic physiological condition, at least during the time span we observed. Therefore, many biomedical studies with MSM mice could be performed using our KO MSM/Ms mice with the benefits of their easier maintenance and handling. They will be available from RIKEN BioResource Center (http://mus.brc.riken.jp/en/) for general research upon request.

## Materials and Methods

### Animals

The MSM/Ms mice used in this study were derived from the colony maintained at the RIKEN BioResource Center. ICR strain mice used as embryo recipients were purchased from CLEA Japan, Inc. (Tokyo, Japan). They were housed under conditions of controlled lighting (daily light period 07:00 to 21:00). All animal experiments described here were approved by the Animal Experimentation Committee at the RIKEN Tsukuba Institute and were performed in accordance with the committee’s guiding principles.

### Preparation of embryos

Fertilized embryos were generated by IVF using female and male MSM/Ms mice at 8 to 20 weeks of age^8^,^9^,^33^. Female mice were injected intraperitoneally with anti-inhibin serum (AIS, 50–100 μl), followed by an injection with human chorionic gonadotropin (hCG, 7.5 IU) 48 h later. The AIS was obtained from an immunized goat as described^8^. Mature metaphase II (MII) oocytes in cumulus were collected from the oviducts 16–17 h after the hCG injection. Spermatozoa were collected freshly from the epididymides of male mice and were preincubated in human tubal fluid (HTF) medium for 1 h at 37 °C under 5% CO_2_ in air. IVF was performed as reported^33^.

### Design of the *nonagouti* targeting vector

Genomic sequences corresponding to the MSM/Ms *nonagouti (a*) locus and the potential off-target sequences (Supplementary Table S4) were retrieved from the ftp site at http://molossinus.lab.nig.ac.jp/msmdb/. The putative exon–intron structure of this gene was determined by the alignment matching the *a* gene of the C57BL/6 mouse strain. The three-target single-guide (sg) RNA sequences of the MSM/Ms *a* gene corresponds to the boundary region of exon 1 and intron 1 (designated as CRISPR #1), exon 3 (CRISPR #2), and exon 4 (CRISPR #3) (Fig. 1a). They were subjected to the annealing reaction of each oligonucleotide and then introduced into the plasmid pX330 into the *Bbs*I restriction enzyme site (http://www.addgene.org/42230/). To evaluate the genome editing efficiency of each vector, a single-strand annealing assay using a pCAG-EGxxFP plasmid was carried out according to Mashiko *et al*.^16^. The genomic fragments of the MSM/Ms *a* gene containing the sgRNA target sequence were amplified by PCR with a cycling programme of 94 °C for 3 min and 35 cycles of 94 °C for 30 s, 60 °C for 30 s, and 72 °C for 30 s and placed between enhanced green fluorescent protein (EGFP) fragments at the multiple cloning site (*Bam*HI–*Eco*RI) of pCAG-EGxxFP. The primers and oligonucleotides used are listed in Supplementary Table S5. Ten-microgram aliquots of the pCAG-EGxxFP-target were mixed with 10 μg of pX330 with or without the sgRNA sequence and then introduced with a NEPA21 electroporator (Nepa Gene Co., Ltd., Chiba, Japan) into 3 × 10^5^ human embryonic kidney (HEK) 293 cells/well cultured in 6-well plates^16^. Plasmids pX330-*Cetn1* and pCAG-EGxxFP-*Cetn1* were used for positive control^16^. The efficiency of DSB-mediated homology-dependent repair was validated by observing EGFP fluorescence at 48 h after transfection. The EGFP fluorescence was observed and quantified using a fluorescence microscope (model BZ-9000; Keyence, Osaka, Japan) and a BZ-II Analyzer image analysis system (Keyence).

### Generation of *nonagouti* KO mice

IVF-derived pronuclear stage embryos were injected with plasmids at a concentration of 5 ng/μl. The injected embryos were cultured in potassium simplex optimized medium (KSOM) at 37 °C under 5% CO_2_ in air. Embryos that reached the 2-cell stage after 24 h of culture were transferred into the oviducts of pseudopregnant ICR female mice that had each been mated with a vasectomized male mouse 1 day before. Equal numbers of ICR embryos were co-transferred with MSM embryos bilaterally. MSM offspring could be identified by their black eye colour irrespective of the *a* allele mutations. Recipient female mice were injected subcutaneously with 1 or 2 mg/kg cyclosporin A (Sigma-Aldrich, St Louis, MO, USA) on day 5. On days 19 or 20, the recipients were examined for the presence of foetuses, and live pups obtained by caesarean section were nursed by lactating ICR strain foster mothers^8^. For genotyping of mice, their tail tips were collected and genomic DNAs were extracted and sequenced. Approximately 500–700 bp genomic fragments containing the target site were amplified by PCR and sequenced using primers listed in Supplementary Table S5. After confirmation of an indel mutation at the target site, PCR fragments were subcloned into a pT7 Blue T-vector (Merck Millipore, Darmstadt, Germany) and sequenced for the determination of each allele. For the alleles A and D, genomic PCR was applied to detect the mutations with primer sets listed in Supplementary Table S5. To determine whether the targeting vector was integrated into the genome of KO lines, we performed genomic PCR using primer sets for the *Cas9* gene sequence, as listed in Supplementary Table S5.

### Analyses of physiological and morphological parameters

At 8 weeks of age, animals were examined using a modified SHIRPA protocol to evaluate their characteristic behaviours and morphologies. Fifty-three items covering various aspects of mouse behaviour and morphologies and sensory responses were tested by visual inspection and manual handling using simple equipment^34^. At 19 weeks of age, animals were subjected to tests for conventional serum biochemical parameters and adipocytokine values. At 26 weeks, the animals were euthanized with an overdose of pentobarbital and examined by macroscopic observations including measurements of organ weights.

### Behavioural analysis

Animals were subjected to the following behavioural tests: light/dark transition test (8 weeks), open-field test (9 weeks), open-field test (10 weeks), home cage activity test (10–11 weeks) according to the methods described on the website of the Japan Mouse Clinic, RIKEN (http://ja.brc.riken.jp/lab/jmc/mouse_clinic/en/). In these tests, female mice were not evaluated because of the possible effects of oestrous cycles on the behaviours measured.

### Tameness test

At 6 weeks of age, animals were examined for tameness. The stay-on-hand test was performed as reported^35^. A researcher picked up the mouse gently and placed it on his/her hand. The durations that the mouse remained on the hand for the three trials were measured and their sums were recorded as the trait data. The maximum time was set to 10s for each trial^35^.

### Microarray analysis

For the microarray analysis, midbrain tissues were isolated manually from mice at 8–9 weeks of age under a dissecting microscope. Total RNA was purified from dissected midbrains using Trizol (Thermo Fisher Scientific, Waltham, MA, USA). Purified total RNA was amplified and labelled with Cy3 using a Low-Input QuickAmp Labeling Kit (Agilent Technologies, Santa Clara, CA, USA). Cy3-labelled cRNAs were hybridized to SurePrint G3 Mouse Gene Expression v2 8 × 60 K Microarray Kits (G4852B; Agilent Technologies) at 65 °C for 17 h. After washing, the hybridized slides were scanned with a DNA microarray scanner (Agilent Technologies). The scanned images were processed with Feature Extraction software (Agilent Technologies) to extract the signal intensities of each probe. The extracted signal data were imported into Gene Spring GX 13.1.1 software (Agilent Technologies) and normalized using the default settings. Probes with a raw signal intensity level less than 100 were excluded from the datasets to remove unreliable noise.

### Pathway and GO analyses

We performed pathway and GO analyses with KEGG and DAVID Bioinformatics Resources 6.7 (https://david.ncifcrf.gov), respectively. Twofold up- and down-regulated genes in KO mice were used for these analyses. *P* < 0.05 was considered statistically significant.

### RT-qPCR analysis

Synthesis of cDNA was performed from the total RNAs of midbrain tissues using the SuperScript^TM^ III First-Strand Synthesis System (Invitrogen, Carlsbad, CA, USA). Prepared cDNA samples were amplified and analysed by RT-qPCR. The primers used are described in Supplementary Table S5. Amplifications were run in a 7900HT Sequence Detector system (Applied Biosystems, Foster City, CA, USA).

### Chromatin immunoprecipitation (ChIP) analysis

ChIP analysis was performed using midbrain tissues as reported^36^. Fixation and isolation of the nuclei of approximately 50 mg of midbrain tissues were performed using truChIP Tissue Chromatin Shearing Reagent kits (Covaris, Woburn, MA, USA). Chromatin shearing was performed using a Covaris S220 instrument. For binding antibodies to magnetic beads, each antibody was incubated with Dynabeads Protein G (Invitrogen) in a ChIP dilution buffer containing 0.01% SDS, 1.1% w/v TritonX-100, 1.2 mM EDTA, and 167 mM NaCl at 4 °C for 1 h with rotation. After washing, the beads were incubated with the chromatin shearing solution at 4 °C for 6 h with rotation. After immunoprecipitation, the beads were washed with a low-salt wash buffer containing 20 mM Tris·HCl, 0.1% SDS, 1% w/v Triton X-100, 2 mM EDTA, and 150 mM NaCl, and a high-salt wash buffer containing 20 mM Tris·HCl, 0.1% SDS, 1% w/v Triton X-100, 2 mM EDTA, and 500 mM NaCl. For elution of DNA from the beads, they were incubated with ChIP direct elution buffer containing 10 mM Tris·HCl, 1% SDS, 5 mM EDTA, and 300 mM NaCl at 4 °C for 15 min with rotation. The solution was incubated at 65 °C overnight, then treated with proteinase K and RNase A and subjected to DNA purification. Prepared DNA samples were amplified and analysed by qPCR. The primers used are described in Supplementary Table S5. Amplifications were run in an ABI 7900HT Sequence Detector (Thermo Fisher Scientific). Relative occupancy values were calculated by determining the immunoprecipitation efficiency (ratios of the amount of immunoprecipitated DNA to that of the input sample).

### Measurement of dopamine concentrations in the midbrain

Dopamine contents in the midbrain were determined using a high-performance liquid chromatography (HPLC) system (EP-300; Eicom, Kyoto, Japan). The midbrain was homogenized with an internal standard, isoproterenol (150 ng/tissue), and after centrifugation, the supernatant was further processed. Dopamine and isoproterenol were separated on SC-50DS columns (diameter, 3.0 mm; length, 150 mm) at 30 °C with a mixture of 100 mM sodium acetate–citric acid buffer (pH 3.5) and methanol (83:17, v/v) containing 190 mg/l sodium 1-octanesulfonate and 5 mg/l EDTA at a flow rate of 0.5 ml/min. Dopamine and isoproterenol levels were measured with an electrochemical detector (ECD-300) with a graphite electrode (WE-3G), using a PowerChrom system (eDAQ, Denistone East, Australia), and dopamine and isoproterenol were determined according to the retention times of standard solutions containing 500 pg each. Amounts of dopamine were quantified by comparison with peak areas of known amounts of isoproterenol.

### Statistical analysis

For the physiological and morphological parameters and tameness tests, the data were analysed by two-way ANOVA analysis (sex × genotype) followed by Tukey multiple comparisons when appropriate. Data from other analyses were subjected to statistical analysis as appropriate and noted in the text. *P* < 0.05 was considered statistically significant.

## Additional Information

**Accession codes:** The microarray data have been deposited in the Gene Expression Omnibus database (GEO; http://www.ncbi.nlm.nih.gov/geo/) and given the series accession number GSE84840.

**How to cite this article:** Hirose, M. *et al*. CRISPR/Cas9-mediated genome editing in wild-derived mice: generation of tamed wild-derived strains by mutation of the *a (nonagouti*) gene. *Sci. Rep.* **7**, 42476; doi: 10.1038/srep42476 (2017).

**Publisher's note:** Springer Nature remains neutral with regard to jurisdictional claims in published maps and institutional affiliations.

## Supplementary Material

Supplementary Information

Supplementary Table S1

Supplementary Table S2

Supplementary Table S3

Supplementary Movie S1

Supplementary Movie S2

## Figures and Tables

**Table 1:** Generation of *nonagouti*-knockout MSM mice by the CRISPR/Cas9 system.

Target (CRISPR construct)	Injected	Cultured (%)	2-cells (%)	Transferred	Pups born (%)	Pups weaned (%)	Homozygous knockout (% per pups)
#1	54	38 (70)	35 (65)	35	0	0	0
#2	39	29 (74)	29 (74)	29	2 (7)	2 (7)	2 (100)
#3	34	28 (82)	28 (82)	28	1 (4)	1 (4)	1 (100)
Total	127	95 (75)	92 (72)	92	3 (3)*	3 (3)	3 (100)

*All male pups.
