# Supplementary material for: CRISPR/Cas9-mediated genome editing in wild-derived mice: generation of tamed wild-derived strains by mutation of the a (nonagouti) gene
Source: Sci Rep. 2017 Feb 14;7:42476. doi: 10.1038/srep42476 (PMC5307340; doi:10.1038/srep42476)
Supplement: Supplementary Information [file srep42476-s1.pdf]

Supplementary Information

CRISPR/Cas9-mediated genome editing in wild-derived mice: generation of tamed wild-derived strains by mutation of the *a* (*nonagouti*) gene

Michiko Hirose, Ayumi Hasegawa, Keiji Mochida, Shogo Matoba, Yuki Hatanaka, Kimiko Inoue, Tatsuhiko Goto, Hideki Kaneda, Ikuko Yamada, Tamio Furuse, Kuniya Abe, Yoshihisa Uenoyama, Hiroko Tsukamura, Shigeharu Wakana, Arata Honda, Atsuo Ogura

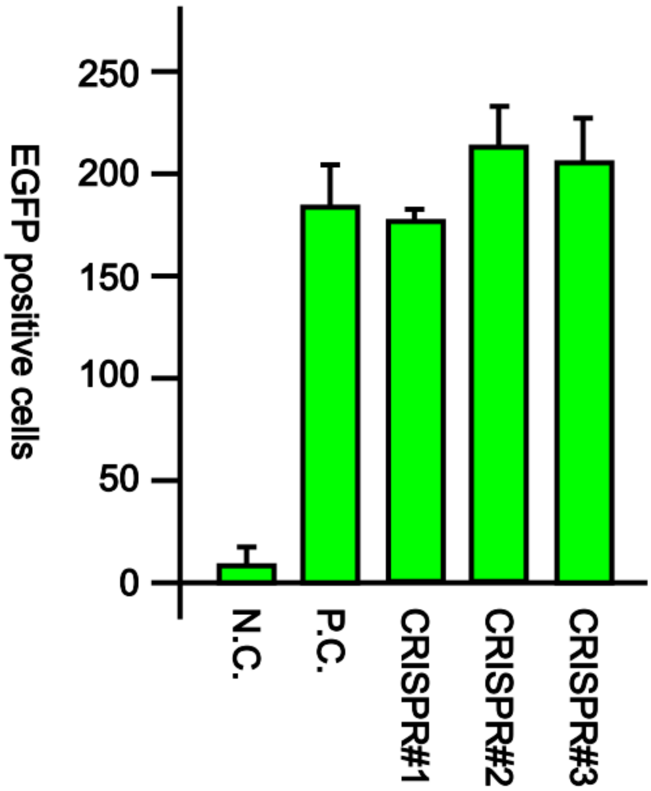

Figure S1. Evaluation of sgRNA sequences for disrupting the *a* (*nonagouti*) gene. Quantitative representation of the DSB efficiency of each sgRNA sequence. Data are shown as the mean ±S.D. N.C., normal control pX330 without an sgRNA sequence; P.C., positive control pX330 with a Cetn1 sgRNS sequence16; CRISPR #1-CRISPR#3, pX330 plasmid with a (nonagouti) target sequences, respectively.

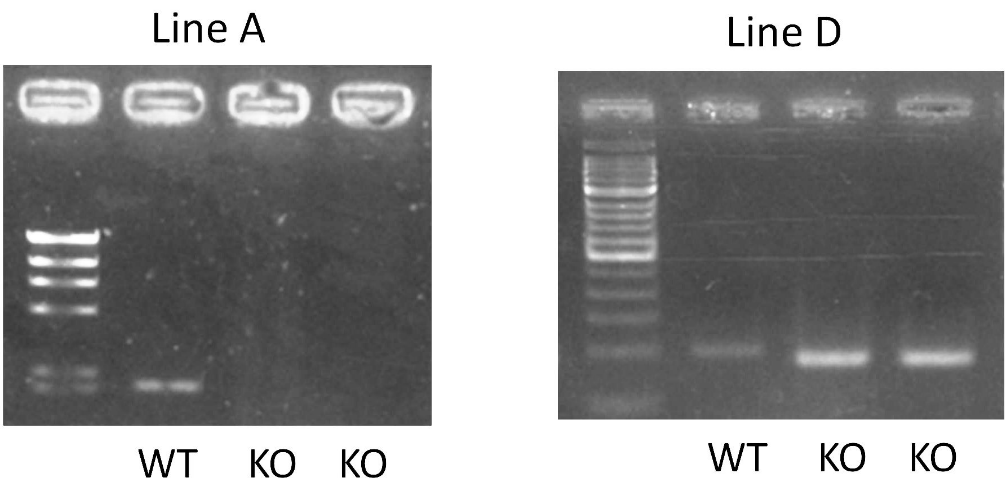

Figure S2. Genomic PCR analysis for identification of mutations in Lines A and D. In Line A, the mutation can be detected by the loss of the PCR product amplified with a primer pair designed for the target site. In Line D, the mutation is identified based on the length of the PCR product that covers the entire the deleted region. The PCR product of Line D mice is 11 bp shorter than that of wild type mice (see Fig. 1C). WT, wild type; KO, knockout.

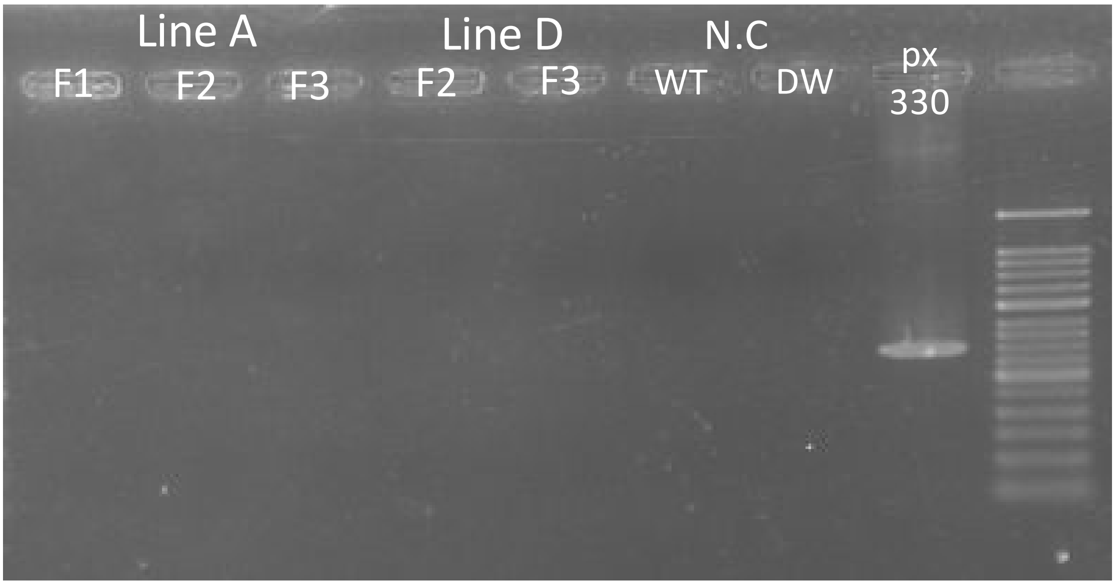

Figure S3. Genomic PCR analysis for detection of the plasmid sequence (Cas9 region) in KO mice from Lines A and D at the F1 to F3 generations. No integration of the plasmid was found in all the mice analyzed. N.C., negative control; WT, wild type; DW, distilled water; px330; the original vector containing Cas9 sequence.

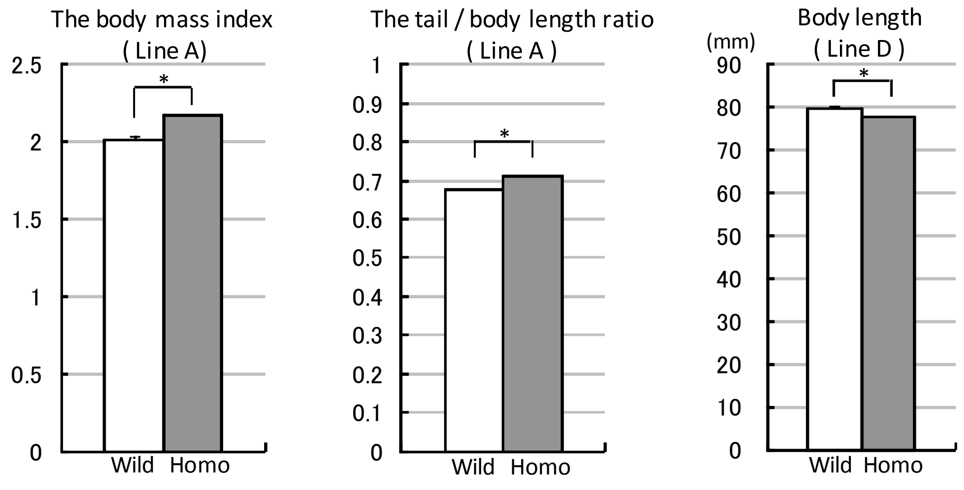

Figure S4. The SHIRPA parameters that showed significant differences between wild-type (WT) and nonagouti homozygous knockout (KO) mice in lines A or D: the body mass index and the tail/body length ratio in line A and body length in line D ($P < 0.05$). No significant differences were found for other parameters. The data were analysed by two-way ANOVA analysis (sex × genotype) followed by Tukey multiple comparisons. Data are presented as the mean±S.E.M.

a

General parameter	Adipocytokine
Aspartate aminotransferase	Monocyte chemotactic protein-1
Lactic dehydrogenase	Inteleukin-6
Alanine aminotransferase	Tumor necrosis factor-alpha
Alkaline phosphatase	Plasminogen activator inhibitor-1
Amylase	Resistin
Total protein	Adiponectin
Glucose	Insulin
Total bilirubin	Amylin
High-density lipoprotein cholesterol	Leptin
Low-density lipoprotein cholesterol	Glucagon-like peptide-1
Total cholesterol	
Triglyceride	
Hemoglobin A1c	

b

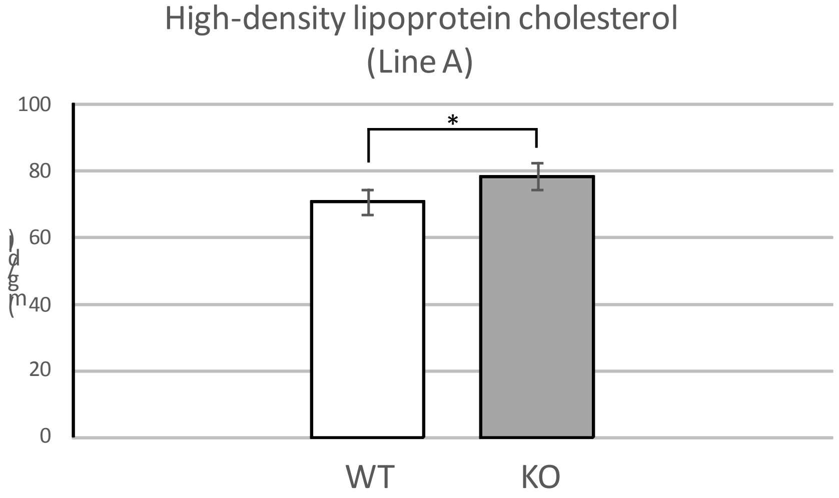

Figure S5. List of serum biochemical parameters analysed in lines A and D. Only the high-density lipoprotein (HDL) cholesterol level in homozygous KO female mice of line A was significantly higher compared with that of the WT litter-mates (**P*** **< 0.05). The data were analysed by two-way ANOVA analysis (sex × genotype) followed by Tukey multiple comparisons. Data are presented as the mean ± S.E.M.**

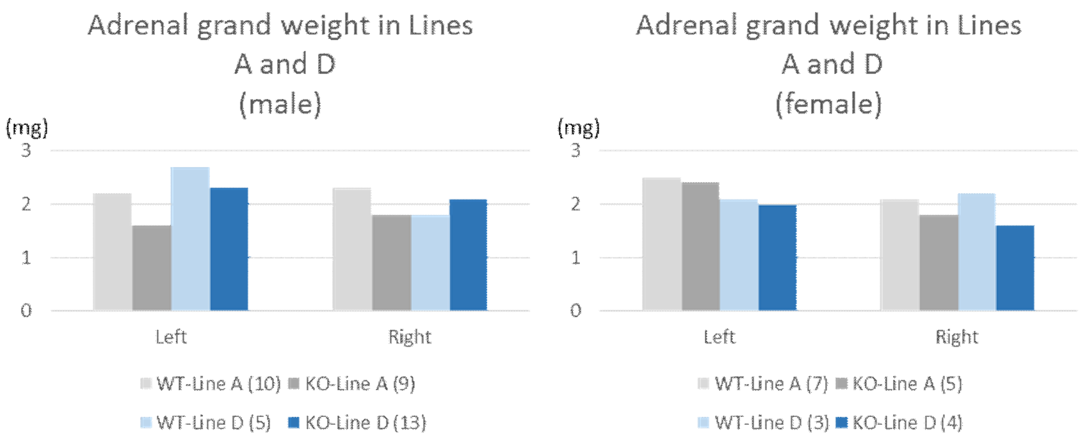

Figure S6. Adrenal gland weights in KO mouse in lines A and D. There was no genotype-related significance. Data are presented as the mean±S.E.M. The numbers in parentheses indicate the number of animals used for each group.

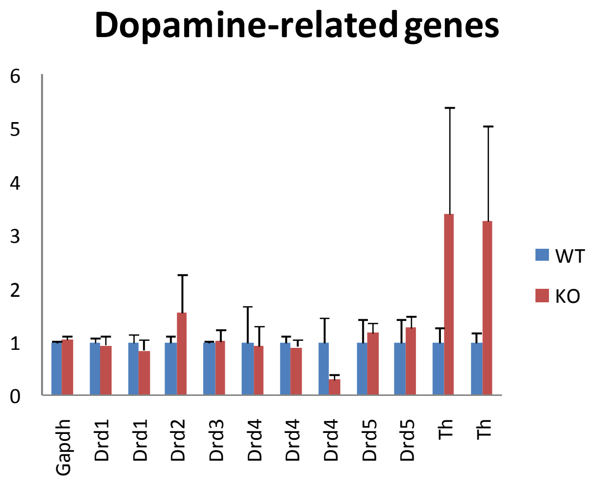

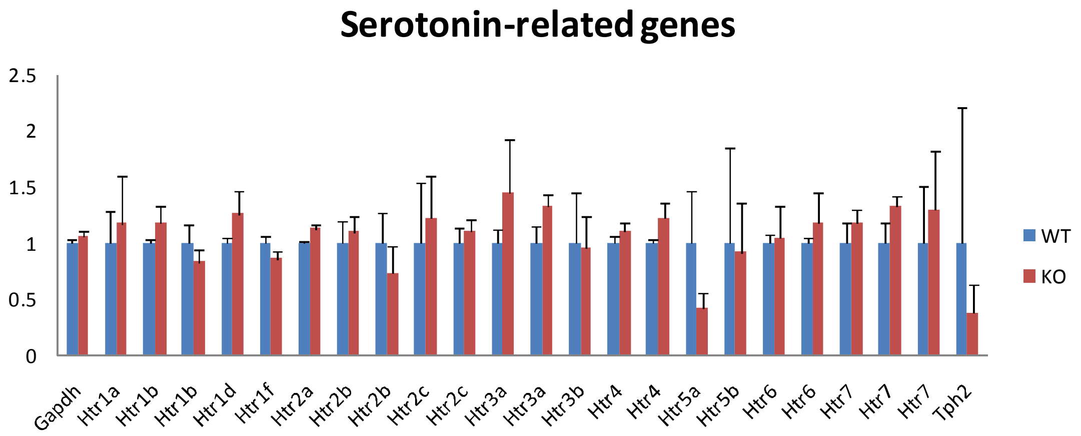

Figure S7. Microarray data for the expression levels of gene related to neurotransmitters in the midbrain. Data are presented as the mean ± S.D. WT: n = 2, KO: n = 6.

Supplementary Movie S1. A WT mouse during the stay-on-hand test. It stayed on the experimenter's hand for less than 1 s.

Supplementary Movie S2. A nonagouti homozygous KO mouse during the stay-on-hand test. It stayed on the experimenter's hand for a longer time than did the WT mice.

Table S4. Potential off target sequences

GCAGAAAAGAAGCCGAGAAG	(CRISPR#2)		Gene Symbol	Gene Name
GCAGAAAAGAAG**gg**GAGAAG	chr.1	intergenic		
GCAGAA**g**AGA**g**GCCGAGAA**t**	chr.3	intergenic		
GCAGAAAAG**t**AGC**a**GAGAAG	chr.4	intergenic		
GCAGAAAAGAAGC**a**GAGAAG	chr.6	intergenic		
GCAGAAA**g**GAAGC**t**GAGAAG	chr.7	genic	Dlg2	discs, large homolog 2 (Drosophila)
GCAGAAAAGAAGC**a**GAGAAG	chr.7	intergenic		
GCAGAAAAGAAG**aa**GAGAAG	chr.8	intergenic		
**c**CAGAAAAGAA**t**CCGAGAAG	chr.9	genic	Fam219b	family with sequence similarity 219, member B
GCAGAAAAGAAGC**t**GAGAAG	chr12	genic	Kcnk13	potassium channel, subfamily K, member 13
GCAGAAAAG**g**AGC**t**GAGAAG	chr13	genic	Fars2	phenylalanine-tRNA synthetase 2 (mitochondrial)
G**g**AGAAAAGAAGCCGAGAAG	chr18	intergenic		

Mismatches from the on-target sequence are indicated by bolded lowercase letters.

GAAAAAGGCTTCGATGAAGA	(CRISPR#3)		Gene Symbol	Gene Name
GAAAAAGGCTTCGATG**gt**GA	chr.5	intergenic		
GAAAAAGGCT**g**CGATGAAGA	chr.X	genic	Itih5l-ps	inter-alpha (globulin) inhibitor H5-like, pseudogene

Mismatches from the on-target sequence are indicated by bolded lowercase letters.

Table S5. Primer sets used for PCR in this study.

Name	Forward (5' to 3')	Reverse (5' to 3')
sgRNA of CRISPR1	caccGAGTCACTTGTGCTGTAAGT	aaacACTTACAGCACAAGTGACTC
sgRNA of CRISPR2	caccGCAGAAAAGAAGCCGAGAAG	aaacCTTCTCGGCTTCTTTTCTGC
sgRNA of CRISPR3	caccGAAAAAGGCTTCGATGAAGA	aaacTCTTCATCGAAGCCTTTTTC
pCAG-EGxxFP/for CRISPR1*	ttgaattCATCCTCCCCTAACCTCCAT	ttggatccAGCAGCAAGCAGGAGAGAAG
pCAG-EGxxFP/for CRISPR2*	ttgaattcGCCAGGCTAATCAGAACCTG	ttggatccTGACCAATTTCAGGAATGCTT
pCAG-EGxxFP/for CRISPR3*	ttgaattCAGTACATGAGCATCGCAGG	ttggatccTCCTCGGCAGCCTAAAAATA
Mutant allele A	CTCAGGATGGATGTCACCCG	TCCGCTTCTCGGCTTCTTTTC
Mutant allele D**	AGTCCTCAACGTCTCTCCTACA	GGTGGCTTGCAGCTGTCG
hCas9	CCGAAGAGGTCGTGAAGAAG	TCGCTTTCCAGCTTAGGGTA
Slc6a3	CTGAAGTCTGACGCTGGAGG	TGGTCAGCTGCACTCCATTC
Slc6a3promoter_ChIP	GGGAAGAGTGCCTGAGCAAA	TCCCTGAACAACCAACCTGC
Slc6a3gene body_ChIP	TCCCTGAACAACCAACCTGC	CAGGCTGAAGTAGAGCAGAACA

*These primers were used for the construction of pCAG-EGxxFP vectors and the genotyping of the offspring.

**The mutation was identified by the length of the PCR product.
